# Supplementary material for: Transcriptomic Insights into Mechanisms of Early Seed Maturation in the Garden Pea (Pisum sativum L.)
Source: Cells. 2020 Mar 23;9(3):779. doi: 10.3390/cells9030779 (PMC7140803; doi:10.3390/cells9030779)

# Zhongwan−6(25)

## Global Summary

%DE = 0.78

# genes with fdr < 0.2  = 465  ( 128 + / 337  −)

# genes with fdr < 0.1  = 425  ( 108 + / 317  −)

# genes with fdr < 0.05  = 387  ( 86 + / 301  −)

# genes with fdr < 0.01 = 323  ( 56 + / 267  −)

# genes in genesets = 0

<FC> = 0

<t−score> = −1005.24

<p−value> = 0

<fdr> = 0.22

**Portrait**

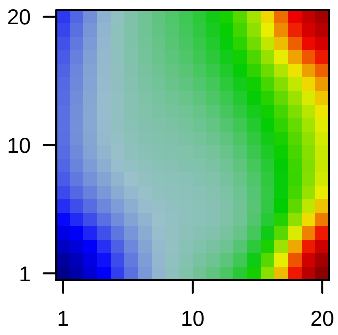


**Top 100 DE genes**

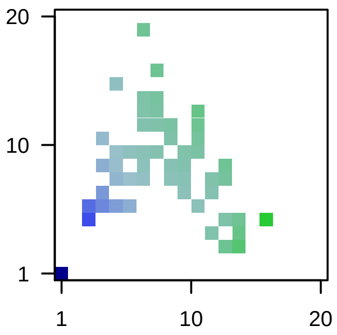


## Global Genelist

| Rank | ID | log(FC) | p−value | fdr | Metagene | Description |
|---|---|---|---|---|---|---|
| 1 | comp26831_c | −0.03 | 3e−15 | 3e−13 | 10 x 8 | |
| 2 | comp9213_c0 | −0.01 | 4e−15 | 3e−13 | 10 x 8 | |
| 3 | comp16669_c | −0.01 | 7e−15 | 3e−13 | 10 x 7 | |
| 4 | TRINITY_DN1 | −0.19 | 8e−15 | 1e−12 | 14 x 3 | |
| 5 | TRINITY_DN3 | −0.12 | 3e−14 | 1e−12 | 14 x 3 | |
| 6 | TRINITY_DN2 | −0.1 | 3e−14 | 9e−12 | 13 x 3 | |
| 7 | comp11051_c | −0.02 | 1e−13 | 9e−12 | 11 x 6 | |
| 8 | comp17299_c | −0.02 | 2e−13 | 6e−11 | 10 x 9 | |
| 9 | comp42763_c | −0.03 | 6e−13 | 6e−11 | 12 x 7 | |
| 10 | TRINITY_DN1 | −0.03 | 2e−12 | 6e−11 | 5 x 15 | |
| 11 | TRINITY_DN1 | −0.22 | 2e−12 | 6e−11 | 16 x 5 | |
| 12 | comp16243_c | −0.01 | 2e−12 | 6e−11 | 6 x 8 | |
| 13 | TRINITY_DN1 | −0.03 | 2e−12 | 6e−11 | 12 x 7 | |
| 14 | TRINITY_DN2 | −0.03 | 3e−12 | 1e−10 | 8 x 16 | |
| 15 | comp15612_c | −0.01 | 3e−12 | 1e−10 | 9 x 12 | |
| 16 | TRINITY_DN5 | −0.03 | 4e−12 | 2e−10 | 13 x 8 | |
| 17 | TRINITY_DN1 | −0.02 | 6e−12 | 4e−10 | 4 x 9 | |
| 18 | TRINITY_DN6 | −0.05 | 9e−12 | 5e−10 | 6 x 6 | |
| 19 | TRINITY_DN3 | −0.03 | 1e−11 | 5e−10 | 11 x 6 | |
| 20 | TRINITY_DN3 | −0.02 | 2e−11 | 5e−10 | 7 x 9 | |

**p−values**

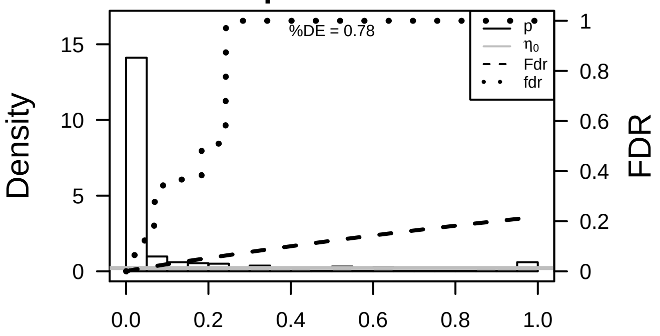

Supplement: Supplementary file 1 [file cells-09-00779-s001.zip › Supplementary materials/FileS3.zip!/FileS2/Hormones - Results/Summary Sheets - Groups/Reports/Zhongwan.6.25..pdf]
